# Supplementary material for: Single-vat single-cure grayscale digital light processing 3D printing of materials with large property difference and high stretchability
Source: Nat Commun. 2023 Mar 6;14:1251. doi: 10.1038/s41467-023-36909-y (PMC9988868; doi:10.1038/s41467-023-36909-y)
Supplement: Supplementary file 1 — Supplementary Information [file 41467_2023_36909_MOESM1_ESM.pdf]

Single-Vat Single-Cure Grayscale Digital Light Processing 3D Printing of Materials with Large Property Difference and High Stretchability

Liang Yue[1], S. Macrae Montgomery[1], Xiaohao Sun[1], Luxia Yu[1], Yuyang Song[2], Tsuyoshi Nomura[3], Masato Tanaka[2], H. Jerry Qi[1,]*

[1]*The George W. Woodruff School of Mechanical Engineering, Georgia Institute of Technology, Atlanta, GA 30332, USA*

[2] *Toyota Research Institute of North America, Toyota Motor North America, Ann Arbor, Michigan, 48105, USA*

[3]*Toyota Central R&D Laboratories, Inc., Bunkyo-ku, Tokyo 112-0004, Japan*

**Corresponding author, Email: qih@me.gatech.edu*

Supplementary Text

Section 1. Grayscale curing kinetics analysis

The conversion of the acrylate reaction is determined from the corresponding absorbance peak intensity of FTIR spectra. The peak intensity at 810 cm^{-1} (I_{810}) associated with the double bond is normalized by the aromatic sp^2 C-H band at 850 cm^{-1} (I_{850}). The degree of conversion (DoC) is calculated by the following equation:

$$p = 1 - \frac{I_{810}/I_{850}}{[I_{810}/I_{850}]_{t=0}} \quad (1)$$

Where *p* represented the DoC.

We use the following frontal photopolymerization (FPP) model[1,2] to study the curing kinetics of the g-DLP ink:

$$z = \frac{\ln\left[\frac{KIt}{-\ln(1-p_C)}\right]}{\mu_e} \quad (2)$$

where *z* represents solidified thickness or curing depth, *K* is the reaction constant, *I* is the light intensity, *t* is the curing time, *Pc* is the DoC at gel point and μ_e is light attenuation coefficient of the resin. Curing time depended depth can be experimentally measured and used to determine *K* and μ_e by fitting equation S2.

Finally, the light dose correlated DoC can be estimated by equations S3[1] with the *K* value to provide a theoretical guideline about curing time and DoC required for g-DLP printing.

$$P=1-\exp(-KIt) \quad (3)$$

Section 2. FEA Simulations

Simulations of the inflatable structures are performed using ABAQUS/Explicit FE package (Dassault Systemes, Vélizy-Villacoublay, France). An explicit analysis is required to use the fluid exchange interaction, which defines a mass flow into the fluid cavity rather than a pressure. The soft regions are assigned a Neo-Hookean material with a Young's modulus of 300kPa, and the stiff regions are assigned a linear elastic material with a Young's modulus of 500MPa. The values of 300kPa and 500MPa are chosen to represent the materials G40 and G70, which are used for printing. This is because the strains in the stiff material remain small enough to be modeled linearly while the extreme strains in the soft regions require a more robust material model. The Neohookean parameters C_{10} and D_1 in ABAQUS are calculated according to the equations provided in the documentation[3]

$$\mu_0 = 2C_{10}, \quad K_0 = \frac{2}{D_1} \tag{4}$$

where μ_0 and K_0 are the initial shear and bulk moduli, respectively. These moduli area calculated from the elastic modulus found using the stress strain curves in Fig. 2 in the main text and the Poisson’s ratio was assumed to be 0.49 since the material is nearly incompressible in the rubbery state.

All regions are meshed with fully integrated shell elements to avoid hourglassing effects. Simulations of the fish fin and heart valve structures are performed in ABAQUS/Standard. These analyses use implicit steps because the fluid exchange interaction is not required. Both soft and stiff materials are modeled as linearly elastic due to the much lower strains compared to the inflation simulations. In the fish fin analysis, the stiff and soft regions are assigned Young's moduli of 500MPa and 100kPa, respectively, and all regions are meshed with C3D10 tetrahedral elements. The value of the G70 material

is reduced to 100kPa for the fish fin simulation to represent the lower effective light dose received due to the thinness of the soft layer. In the heart valve analysis, the stiff and soft regions are assigned Young's moduli of 500MPa and 300kPa, respectively. The properties for the heart valve represent the grayscale values of G0 and G70, which were used for printing. The soft flaps are meshed with C3D8 elements, and the stiffer base is meshed with C3D10 elements. A uniform pressure load is applied on the inner face of the soft flap, and the deformation is analyzed using a Static, Riks analysis to capture snap-through instability experienced by the soft flaps.

Section 3. Reference resins formulation used in the work

1. Figure 11, control resin without 2-HEA: Isobornyl acrylate (Sigma-Aldrich, MO, USA) and AUD (Ebecryl 8413, Allnex, GA, USA) are mixed with the weight ratio of 3:1. Then 1wt% photoinitiator (Irgacure 819, Sigma-Aldrich, MO, USA) and 0.05wt% photo absorber (Sudan I, Sigma-Aldrich, MO, USA) are added and mixed well.
2. Figure 13, control resin with poor hydrogen bond moieties: Poly(ethylene glycol) diacrylate (PEGDA, Sigma-Aldrich, MO, USA), butyl acrylate (BA, Sigma-Aldrich, MO, USA) and isobornyl acrylate (Sigma-Aldrich, MO, USA) are mixed with the weight ratio of 1:3:1. Then 1wt% photoinitiator (Irgacure 819, Sigma-Aldrich, MO, USA) and 0.05wt% photo absorber (Sudan I, Sigma-Aldrich, MO, USA) are added and mixed well.
3. Figure 21, alternative resin with rich hydrogen bond moieties: 2-[[(Butylamino)carbonyl]oxy]ethyl acrylate (Sigma-Aldrich, MO, USA), acrylate acid (Sigma-Aldrich, MO, USA) and AUD (Ebecryl 8413, Allnex, GA, USA) are mixed

with the weight ratio of 2:2:1. Then 1wt% photoinitiator (Irgacure 819, Sigma-Aldrich) and 0.05wt% photo absorber (Sudan I, Sigma Aldrich) are added and mixed well.

Supplementary Table

Table 1. Detailed grayscale and their corresponding RGB value, light intensity and volumetric energy input.

Grayscale	RGB value	Printer light intensity mW/cm^2	Total energy per volume mJ/mm^3
G0	255	24.4	14.64
G10	229	21.1	12.66
G20	204	17.6	10.56
G30	179	14.3	8.58
G40	153	9.9	5.94
G50	128	5.5	3.3
G60	102	3.2	1.92
G70	77	1.4	0.84

Supplementary Figures

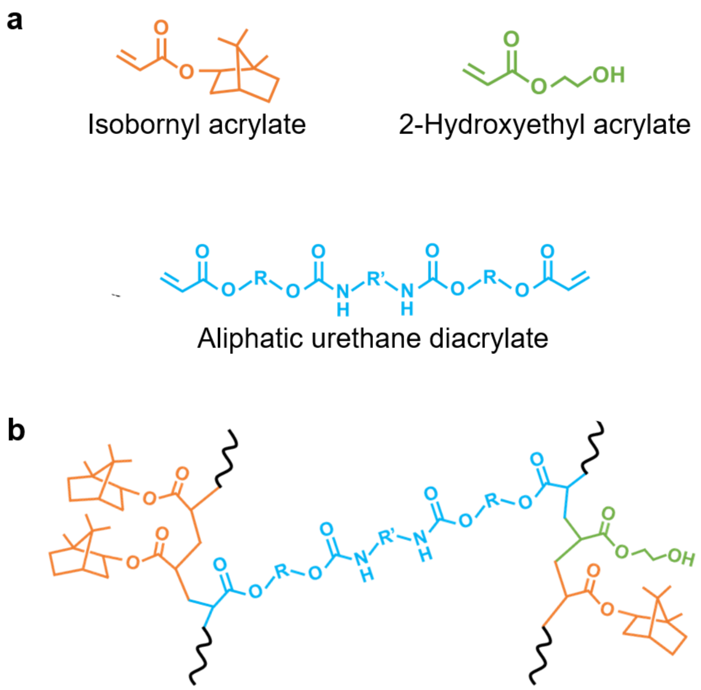

Figure 1. a) Chemical structure of acrylate monomers for the ink; b) Chemical structure of the crosslinked network.

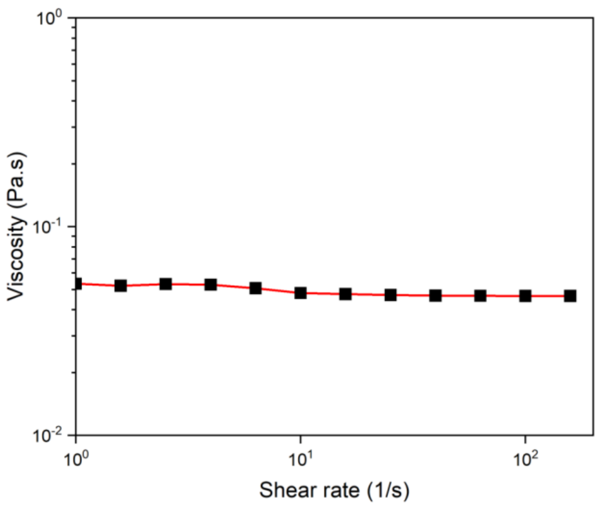

Figure 2. The viscosity of the g-DLP ink was measured with a rotational rheometer (Discovery HR-2, TA Instruments, New Castle, DE, USA) at 25 °C (using a 20-mm-diameter cross-hatched plate, gap height of 0.5 mm).

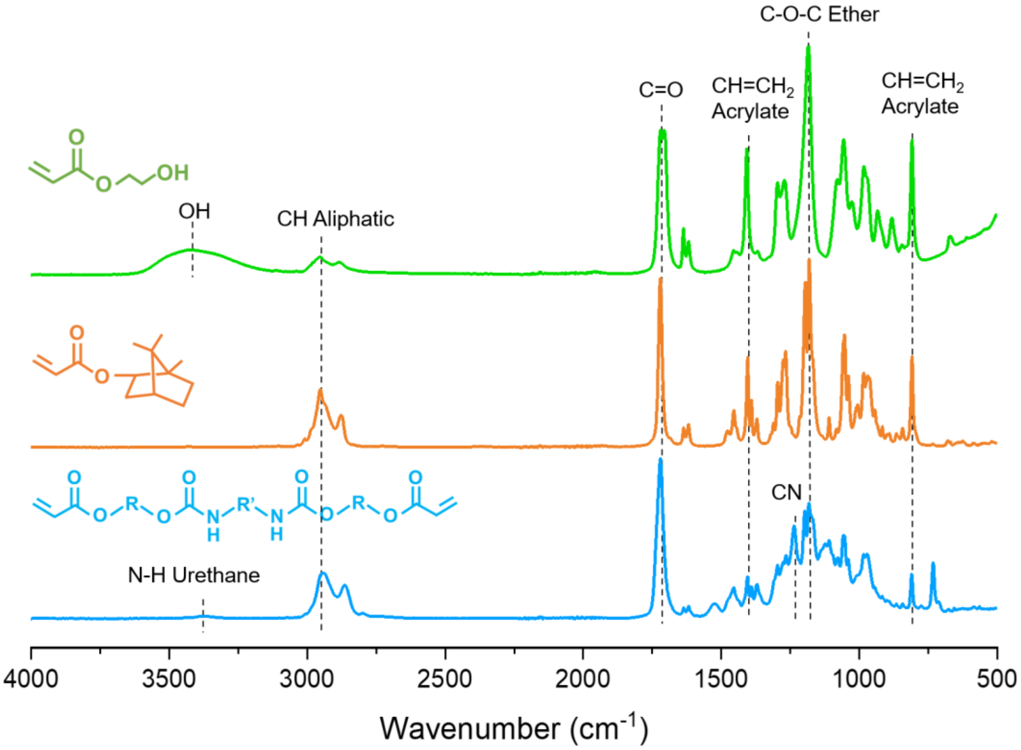

Figure 3. FTIR spectra of the monomers used for g-DLP ink.

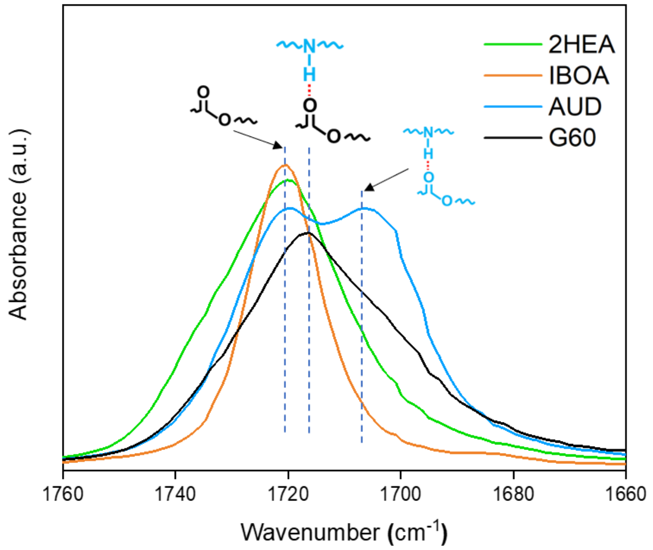

Figure 4. FTIR spectrum at the ranges of 1660-1760 cm^{-1}. The C=O stretching is at 1720 cm^{-1}. The shifting of C=O for G60 was due to the hydrogen bonding associated with the N-H group.

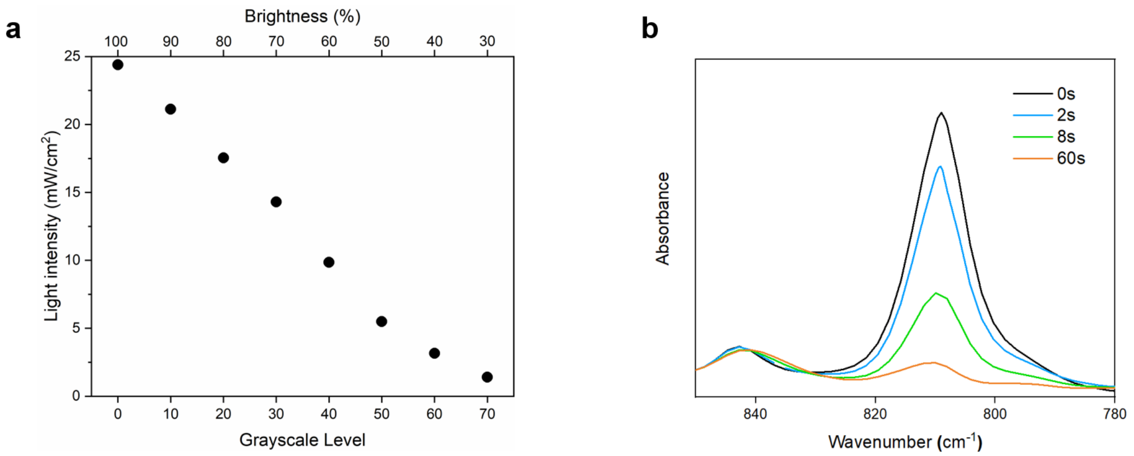

Figure 5. a) UV light intensity as a function of grayscale level and brightness; b) FTIR of C=C peak(809 cm^{-1}) intensity decreasing as the curing time increases under the G50 light intensity (5.5 mW/cm^2).

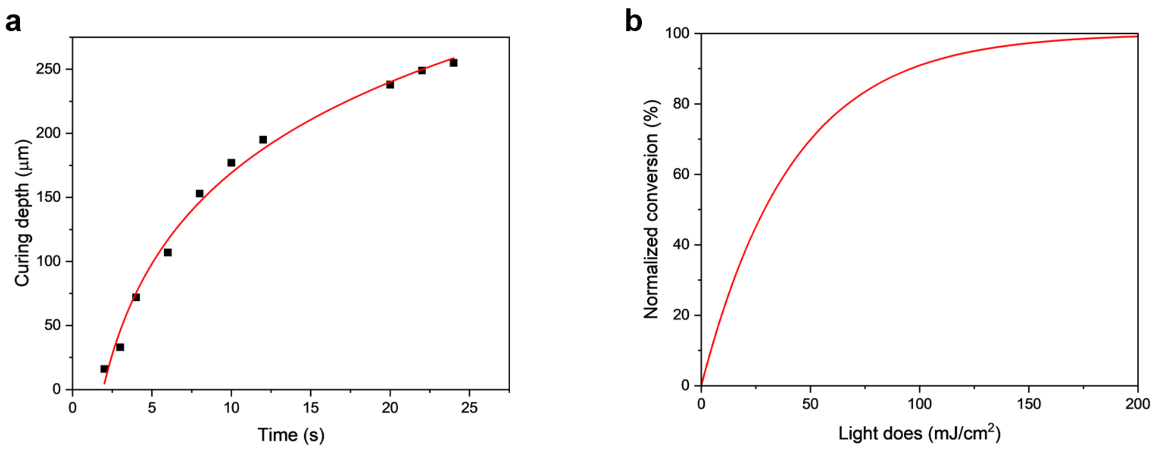

Figure 6. a) The curing depth as a function of curing time for the g-DLP ink using the G50 (5.5 mW/cm^2) light. By fitting the curing depth curve with equation S2, the parameters K and μ_e are determined: K =0.024 cm^2·mW^{-1}·s^{-1}, μ_e= 9.78 mm^{-1}, respectively; b) correlation between DoC and total light does predicted with the determined K by equation S3.

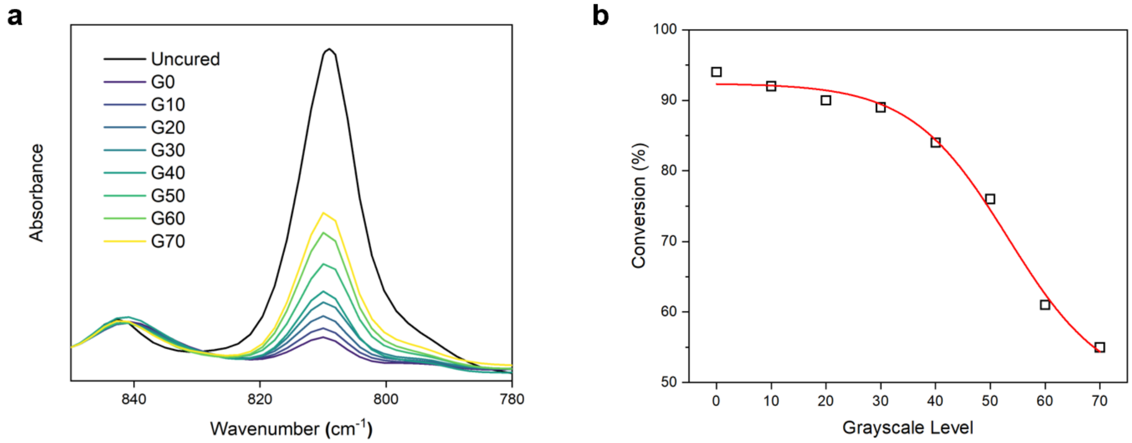

Figure 7. a) FTIR spectra at the ranges of 780-850 cm^{-1} of the acrylate C=C bond printed at different grayscale levels; b) reaction conversion of the acrylate printed at different grayscale levels.

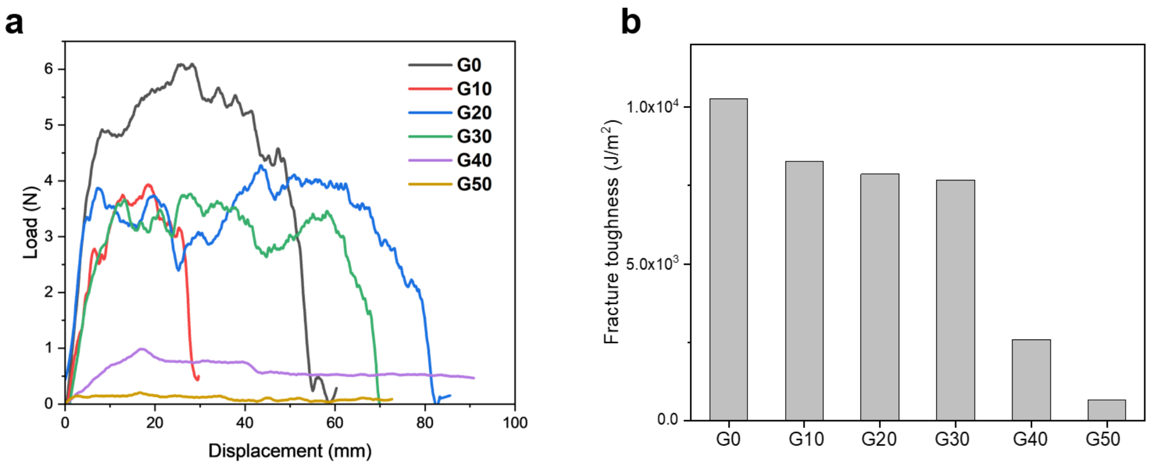

Figure 8. a) The tearing test force-displacement curves of g-DLP printed samples; b) calculated fracture toughness of g-DLP printed samples. The fracture toughness was measured through the tearing test. The rectangular samples of dimensions of 50.0 mm(length) × 7.2 mm(width) were prepared with a precut crack which was 20mm long in the longitudinal direction. Following the reported method[4], two stiff backing layers are attached to the sample surface. When the crack propagates in a steady state, the fracture toughness can be given as 2F/h, where F is the plateau force and h is the thickness of the samples.

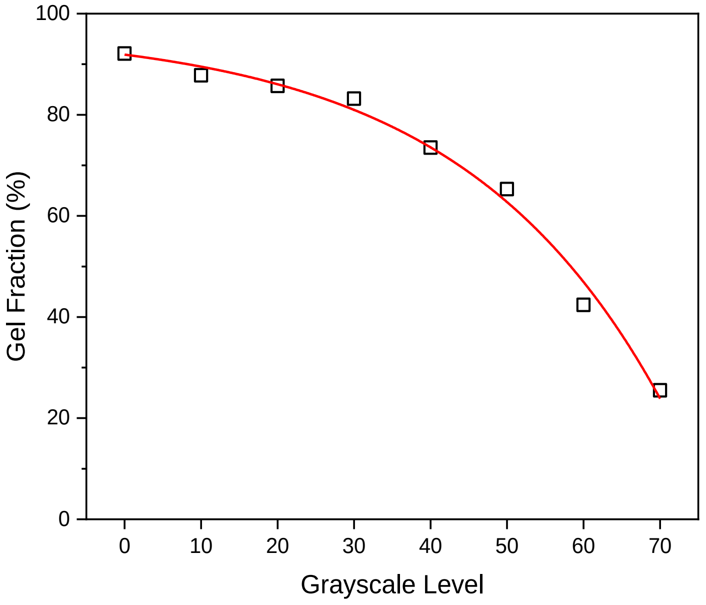

Figure 9. The gel fraction of g-DLP printed samples were measured by soaking in acetone for 24 hours, followed by drying for 24 hours.

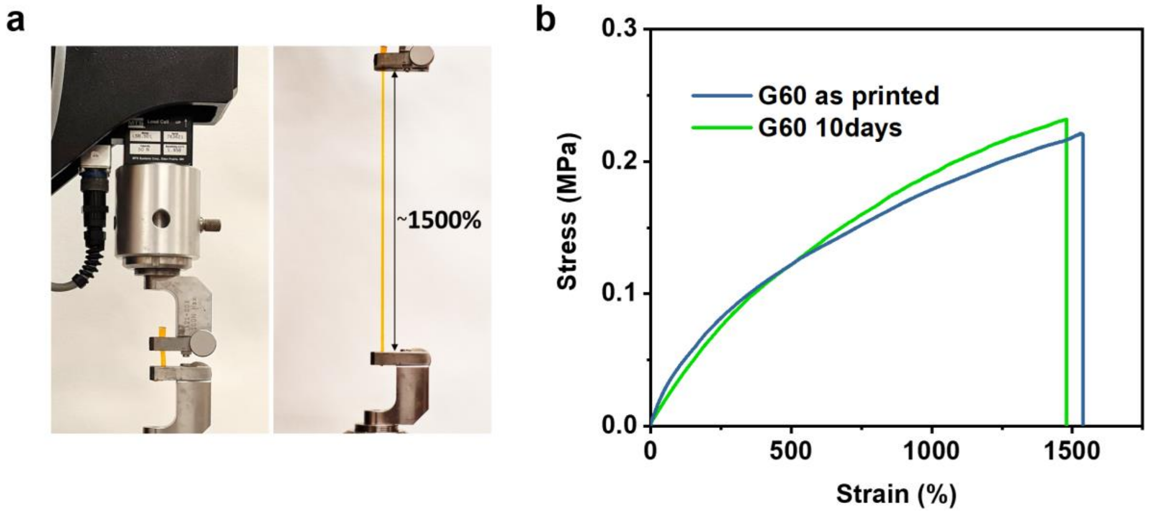

Figure 10. a) Snapshots of G60 tensile test; b) strain-stress curves of G60 samples comparison after 10 days. The printed samples were covered with aluminum foil to avoid light.

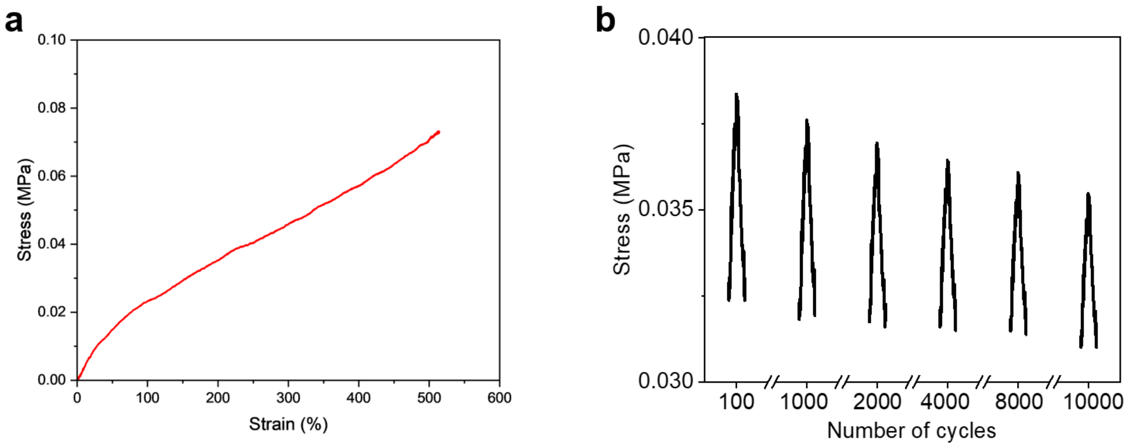

Figure 11. a) Stress-train curves of the control ink with only AUD and IBOA (1:3 wt/wt) at the same conversion with G60 (~60%); b) fatigue test of the control ink with an applied strain between 200 to 300%. Decreasing of the stress indicated network relaxation of the control sample due to less hydrogen bonding.

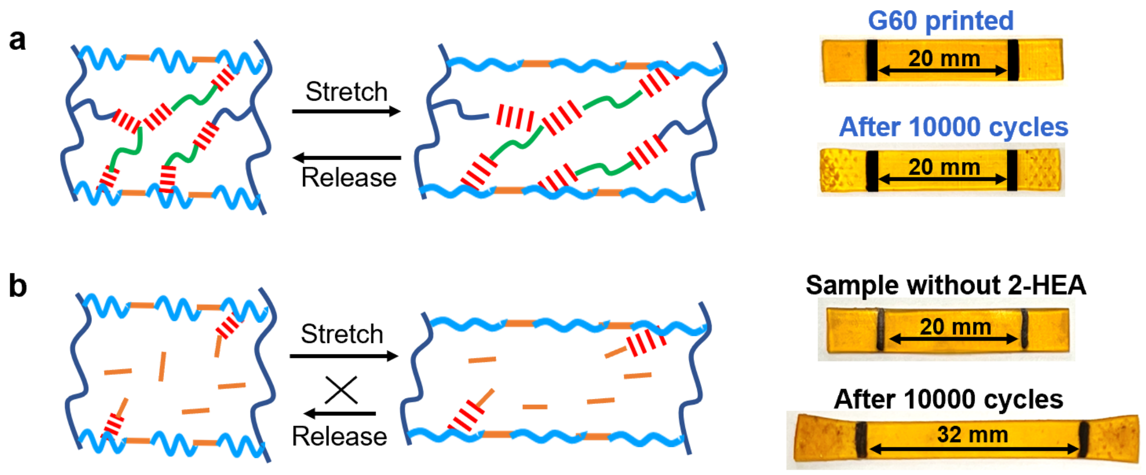

Figure 12. Schematic and photos of a) G60 sample and b) control sample without 2-HEA after 10000 cycles test.

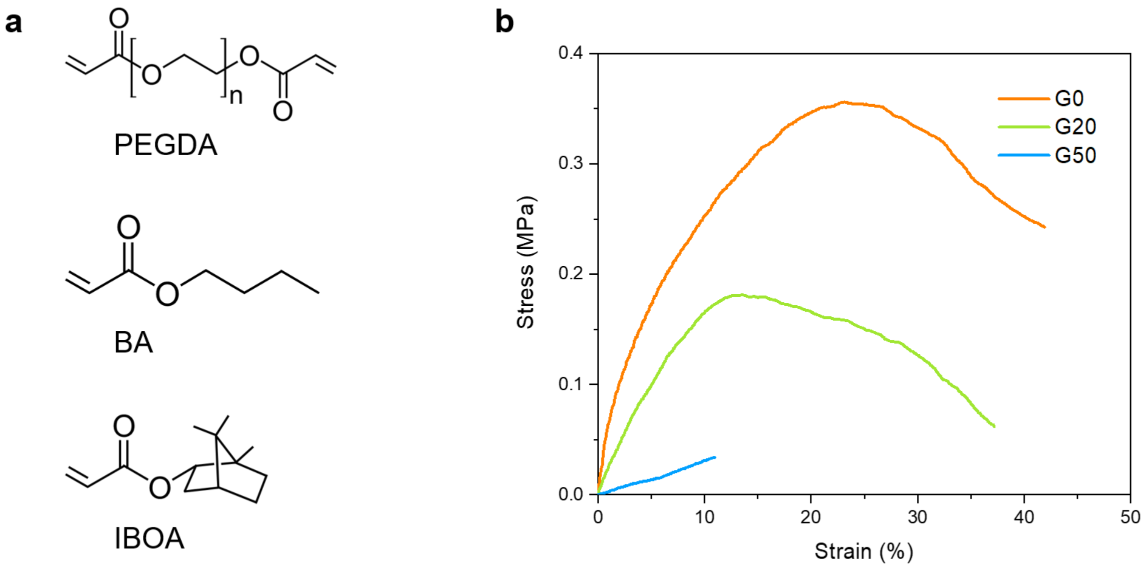

Figure 13. a) a control ink that does not form hydrogen bonding for the g-DLP ink, where the AUD was replaced by poly(ethylene glycol) diacrylate (PEGDA, Sigma-Aldrich, MO, USA) and 2-HEA was replaced by butyl acrylate (BA, Sigma-Aldrich, MO, USA); b) strain-stress of the g-DLP printed samples with the control ink.

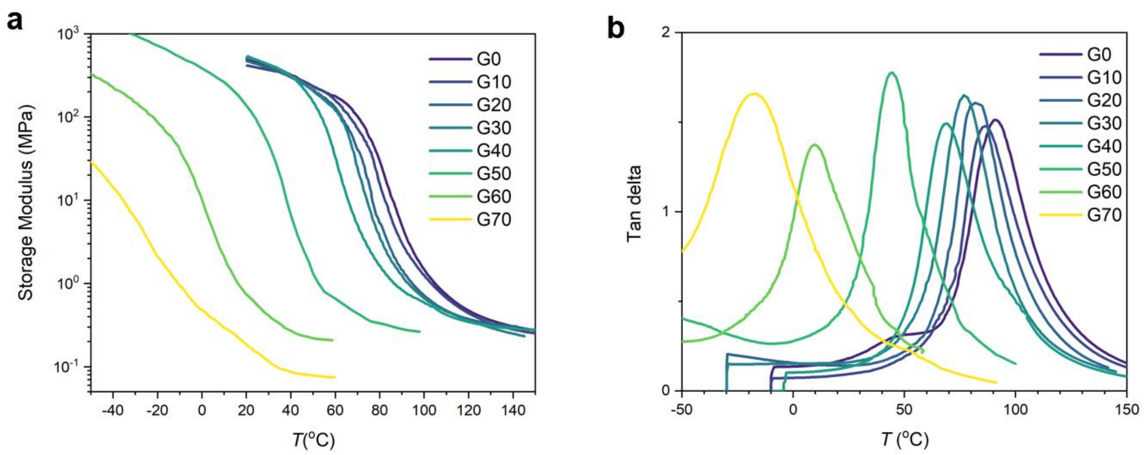

Figure 14. a) Storage modulus and b) tan δ as a function of temperature for the g-DLP printed samples.

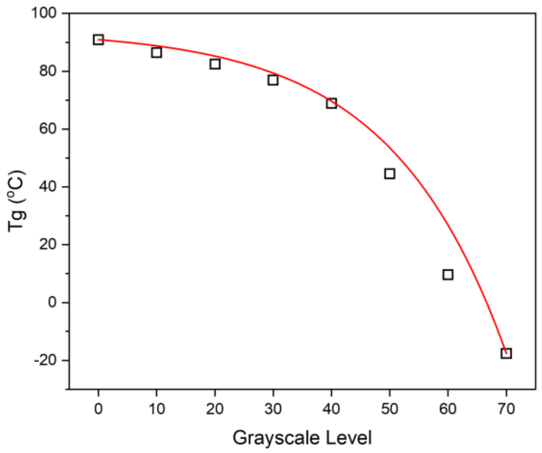

Figure 15. Glass transition temperatures of g-DLP printed samples.

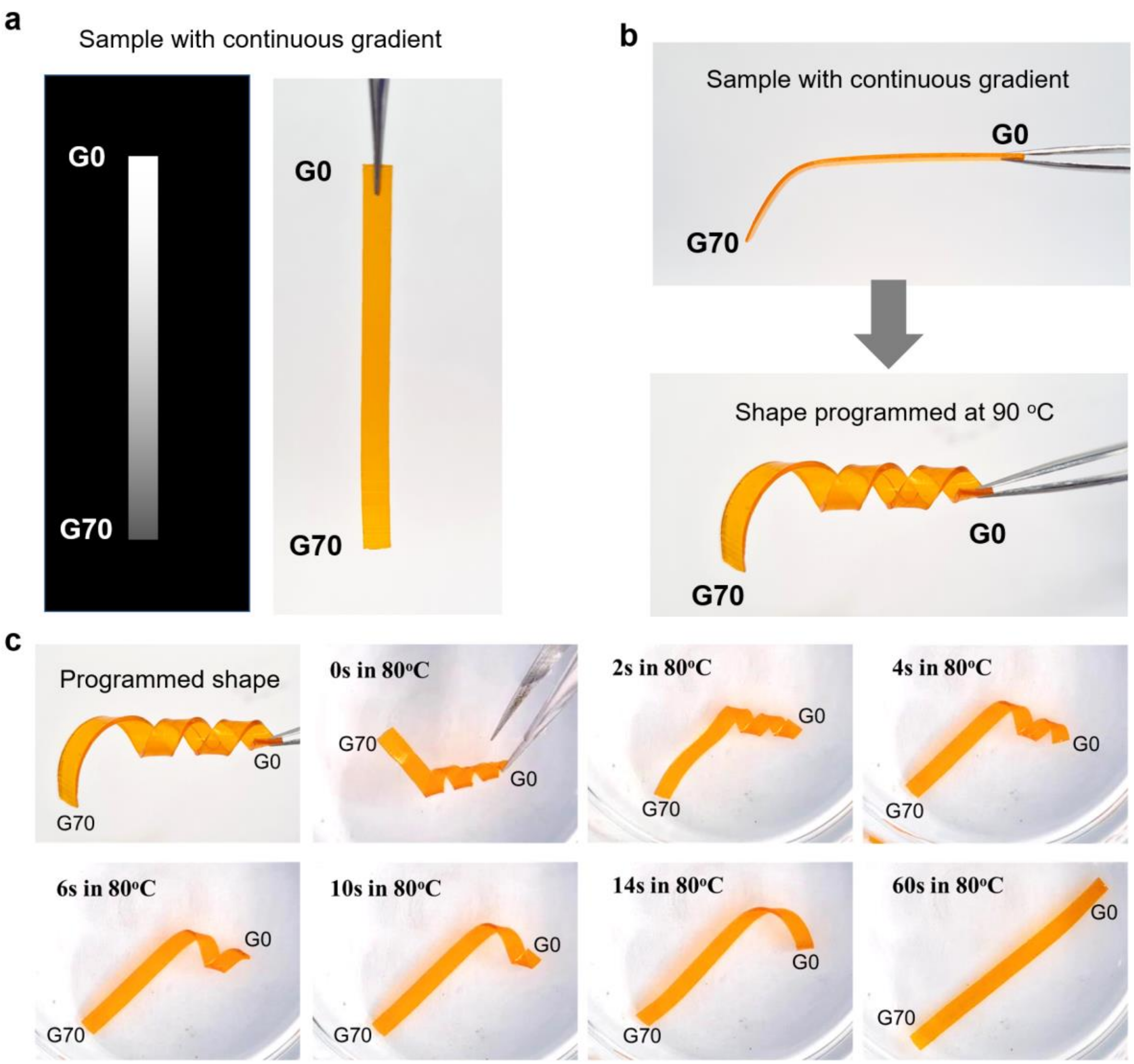

Figure 16. a) g-DLP printed sample with a continuous gradient; b) sample with continuous gradient was programmed into a helix shape at 90°C and fixed by rapid cooling; c) shape recovery of the programmed sample in 80°C water bath.

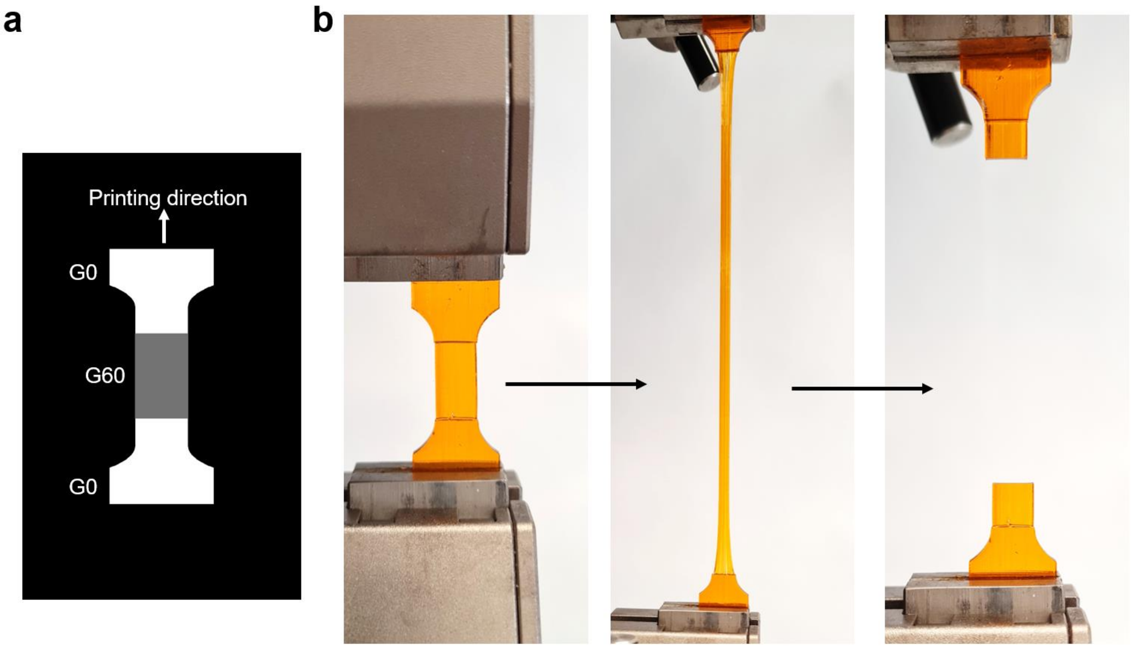

Figure 17. a) Design of a G0 and G60 sample; b) stretching and breaking of g-DLP printed G0 and G60 composite.

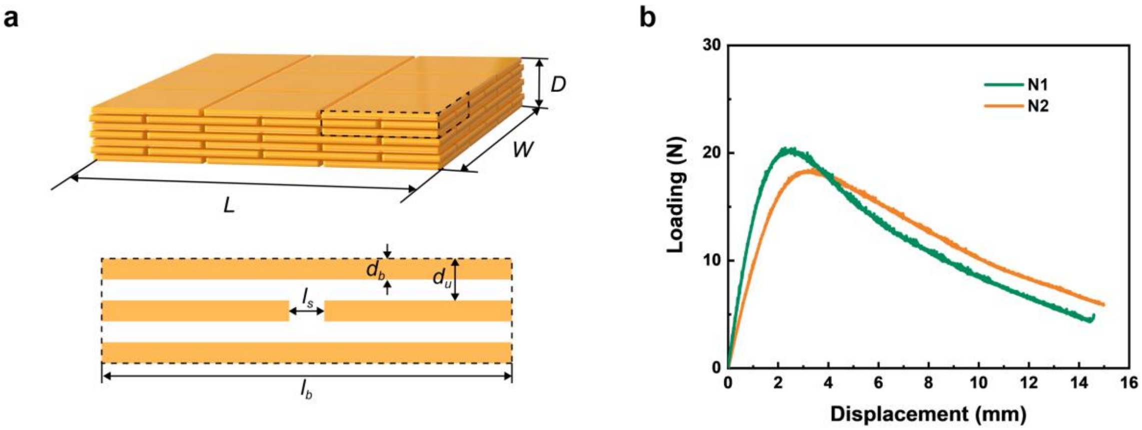

Figure 18. a) Design parameters of g-DLP printed nacre composites; b) properties of nacre composites with different parameters. N1: l_b=2.9mm, d_b=0.1mm, l_s=0.1mm, d_u=0.2mm; N2: l_b=4.7mm, d_b=0.2mm, l_s=0.3mm, d_u=0.4mm.

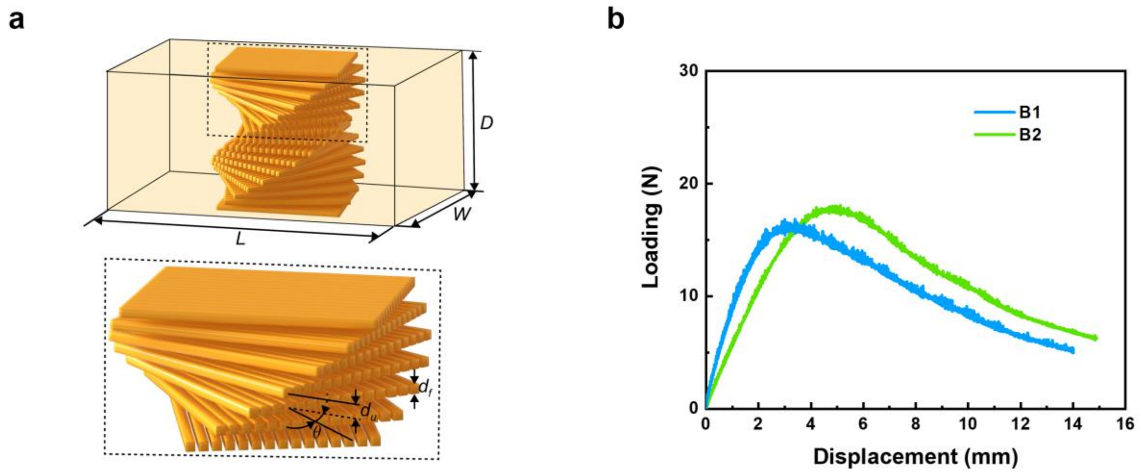

Figure 19. a) Design parameters of g-DLP printed bouligand composites; b) properties of bouligand composites with different parameters. B1: d_f=0.1mm, d_u=0.2mm, θ=30º ; B2: d_f=0.1mm, d_u=0.2mm, θ=12º .

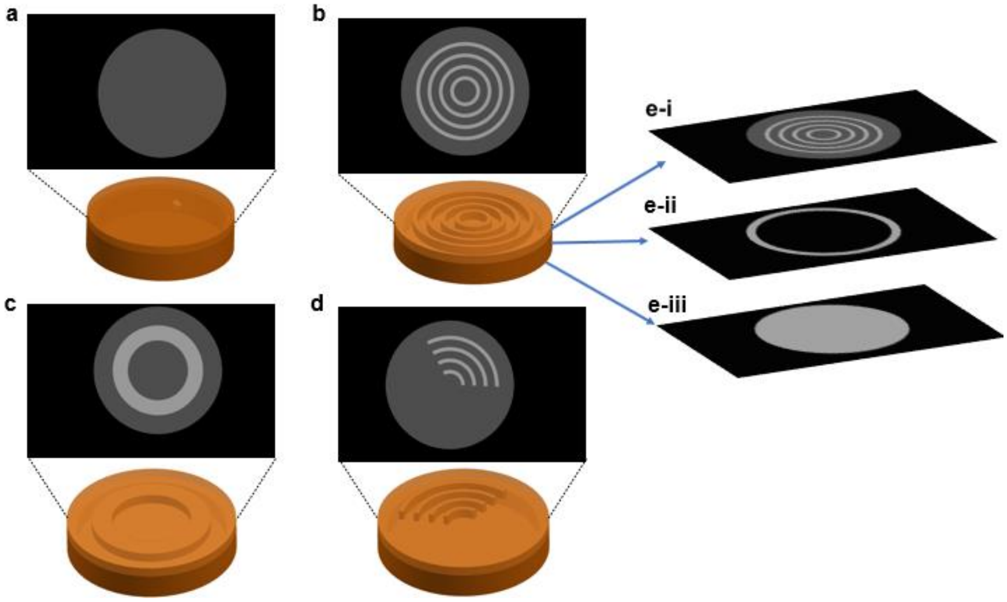

Figure 20. Detailed design of the inflatable box. a-d) the inflatable membrane (G60, gray region) with fibers embedded (G40, bright region); e) i-iii represents the projection images of the membrane, chamber, and bottom of the inflatable box, respectively.

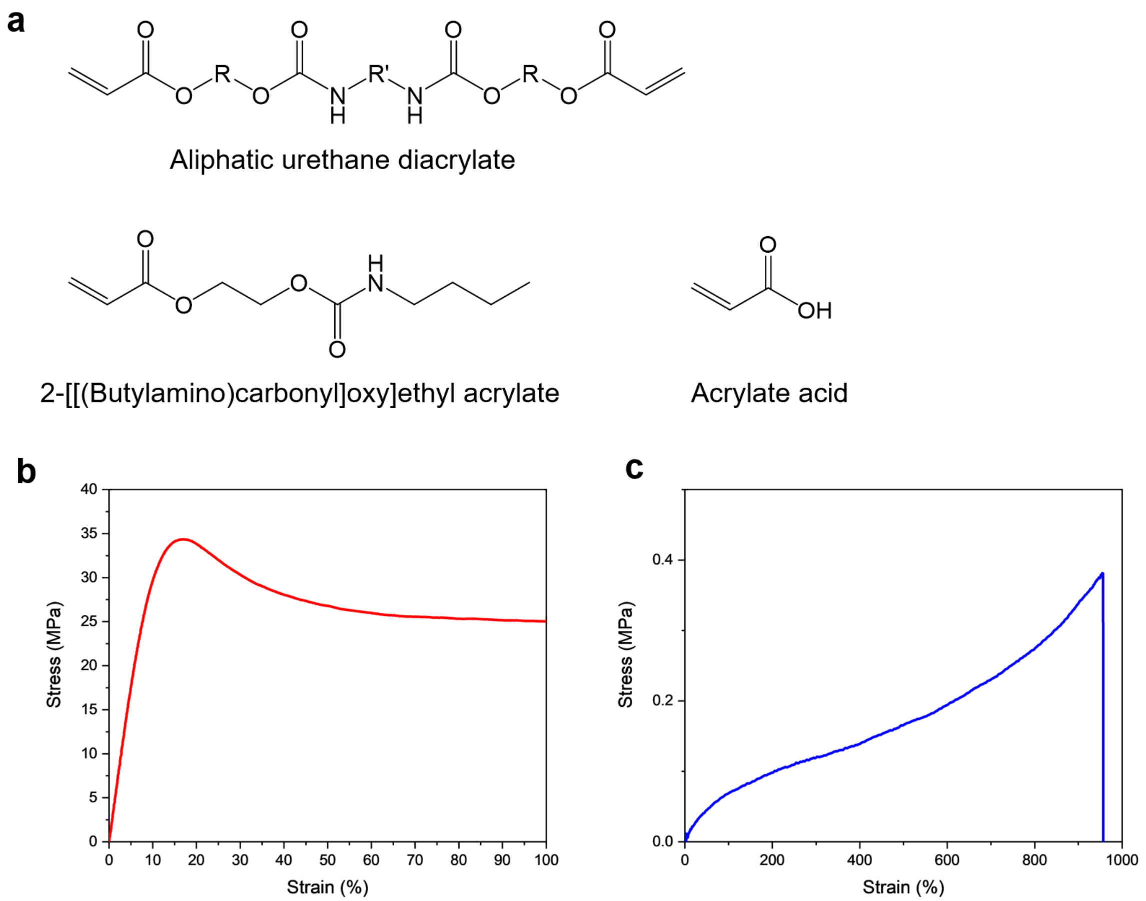

Figure 21. a)Chemical structure of monomers used for exemplary resin formulation; strain-stress curves of g-DLP printed exemplary resin of b) stiff thermoset state printed in G0; c) rubbery organogel state printed in G60.

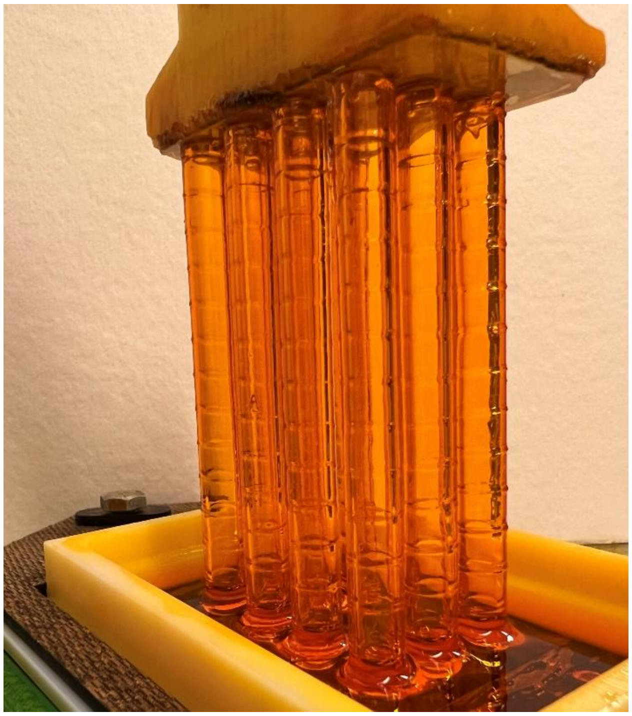

Figure 22. Photo of a g-DLP printing of 12 tentacle-like actuators in one batch, printing speed 1mm/min.

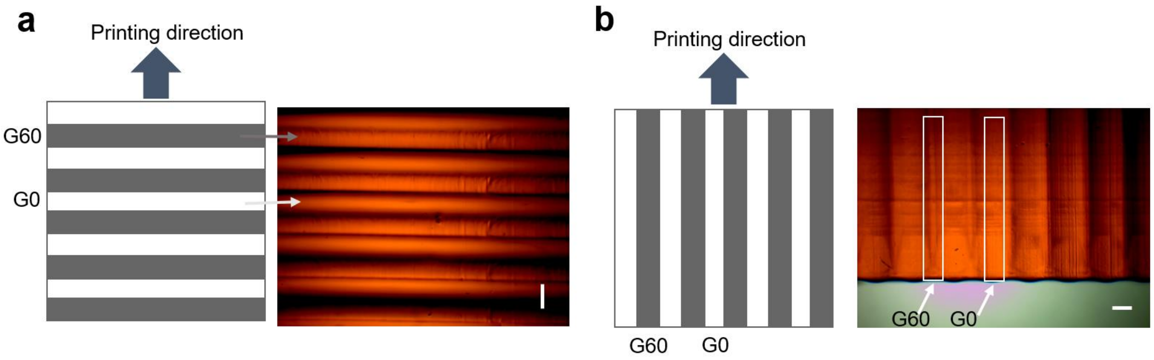

Figure 23. Optical microscope images of g-DLP printed G0 and G60 layered structure with a layer thickness of 200 μm, a) printed in the horizontal direction; b) printed in the vertical direction. The scale bar is 200 μm.

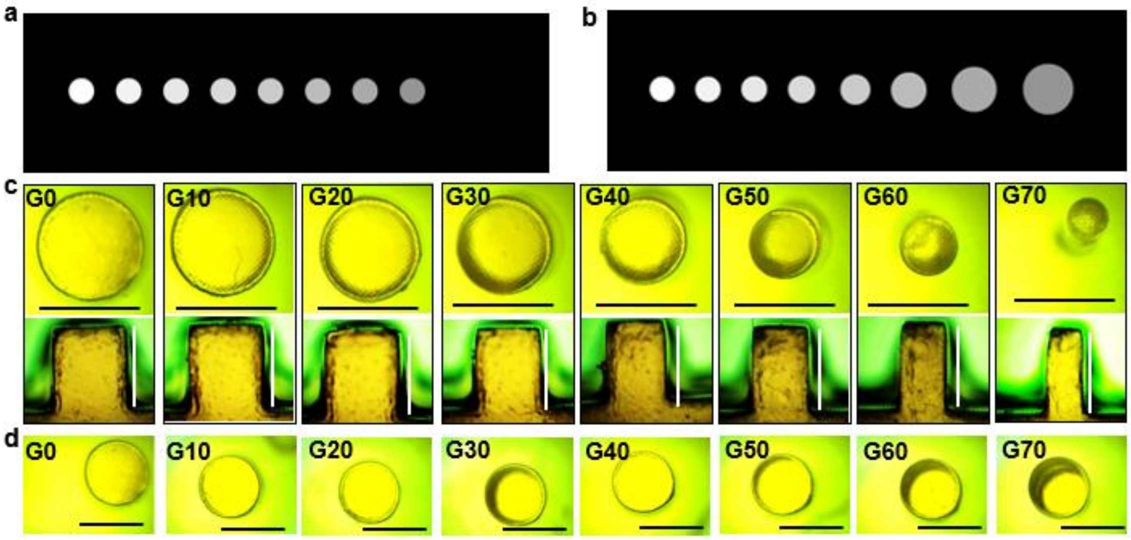

Figure 24. g-DLP printing could result a size distortion on the printing surface, which could decrease the resolution of small structures. Adjusting the projecting size could compensate for such distortion. a) Projection image of designed cylinders from G0 to G70; b) projection images of designed cylinders with compensation; c) size and heights of printed cylinders; d) size of printed cylinders with compensation. All scale bars are 500 µm.

Supplementary References:

1 Wu, J. *et al.* Evolution of material properties during free radical photopolymerization. *Journal of the Mechanics and Physics of Solids* **112**, 25-49 (2018). https://doi.org:https://doi.org/10.1016/j.jmps.2017.11.018

2 Vitale, A., Hennessy, M. G., Matar, O. K. & Cabral, J. T. Interfacial Profile and Propagation of Frontal Photopolymerization Waves. *Macromolecules* **48**, 198-205 (2015). https://doi.org:10.1021/ma5021215

3 *Hyperelastic behavior of rubberlike materials*, <http://130.149.89.49:2080/v2016/books/usb/default.htm?startat=pt05ch22s05abm07.html#usb-mat-chyperelastic> (2015).

4 Bai, R., Yang, J. & Suo, Z. Fatigue of hydrogels. *European Journal of Mechanics - A/Solids* **74**, 337-370 (2019). https://doi.org:https://doi.org/10.1016/j.euromechsol.2018.12.001
