# Supplementary material for: Single-vat single-cure grayscale digital light processing 3D printing of materials with large property difference and high stretchability
Source: Nat Commun. 2023 Mar 6;14:1251. doi: 10.1038/s41467-023-36909-y (PMC9988868; doi:10.1038/s41467-023-36909-y)
Supplement: Supplementary file 3 — Description of Additional Supplementary Files [file 41467_2023_36909_MOESM3_ESM.pdf]

File Name: Supplementary Movie 1
Description: g-DLP printed sample with continuous gradient.

File Name: Supplementary Movie 2
Description: Composite designed with sequential deformation.

File Name: Supplementary Movie 3
Description: Hydrodynamic performance of g-DLP printed heart valve.

File Name: Supplementary Movie 4
Description: g-DLP printed biomimetic fish fin structure.

File Name: Supplementary Movie 5
Description: g-DLP printed pufferfish.

File Name: Supplementary Movie 6
Description: g-DLP printed inflatable membranes.

File Name: Supplementary Movie 7
Description: g-DLP printed pneumatic actuators.

File Name: Supplementary Movie 8
Description: g-DLP printed tentacle actuator.
